# Supplementary material for: Observation of the nonanalytic behavior of optical phonons in monolayer hexagonal boron nitride
Source: Nat Commun. 2024 Mar 2;15:1938. doi: 10.1038/s41467-024-46229-4 (PMC10908826; doi:10.1038/s41467-024-46229-4)
Supplement: Supplementary file 1 — Supplementary Information [file 41467_2024_46229_MOESM1_ESM.pdf]

Supplementary Information for
"Observation of the Nonanalytic Behavior of Optical Phonons in Monolayer Hexagonal Boron Nitride"

Jiade Li[1, 2], Li Wang[1], Yani Wang[3, 4], Zhiyu Tao[1, 2], Weiliang Zhong[1, 2], Zhibin Su[1, 2], Siwei Xue[1], Guangyao Miao[1], Weihua Wang[1], Hailin Peng[3, 4], Jiandong Guo[1, 2*], & Xuetao Zhu[1, 2*]

[1] *Beijing National Laboratory for Condensed Matter Physics and Institute of Physics, Chinese Academy of Sciences, Beijing 100190, China*

[2] *School of Physical Sciences, University of Chinese Academy of Sciences, Beijing 100049, China*

[3] *Center for Nanochemistry, Beijing Science and Engineering Center for Nanocarbons, Beijing National Laboratory for Molecular Sciences, College of Chemistry and Molecular Engineering, Peking University, Beijing 100871, China*

[4] *Beijing Graphene Institute (BGI), Beijing 100095, China*

[*] Jiandong Guo (jdguo@iphy.ac.cn) and Xuetao Zhu (xtzhu@iphy.ac.cn).

Comparison of 2D-HREELS and conventional HREELS

Dispersion measurements in a conventional HREELS system require the change of the scattering geometry by mechanically rotating sample, monochromator, or analyzer. The analyzer collects the scattered electrons at a certain angle, which carries important momentum information of phonons. In our 2D-HREELS system, a hemispherical electron analyzer is employed to simultaneously measure the energies and angle distributions of scattered electrons. Without rotating sample, monochromator, or analyzer, a HREELS spectrum can be scanned for a certain direction through the BZ in a single measurement, and thus a momentum-dependent spectral intensity distribution can be directly obtained. In Fig. S1, we illustrate a comparison between our 2D-HREELS and conventional HREELS measurements.

Origin of replica signals in HREELS spectra

In this section, we will demonstrate that the replica signals originate from the roughness of the h-BN/Cu foil surface. The h-BN grown Cu foil is rough due to the surface roughening of Cu foil [Fig. S2a]. Considering the incident beam size of our HREELS is around 1 mm, the scattering geometry cannot be perfect on a rough surface. As shown in Fig. S2b, according to Eq. (2)-(3) in Methods, if the sample surface is atomically flat, for a specific θ_i, a specific q occurs only at a specific θ_s, that is, one-to-one correspondence between q and θ_s. In this case, the true momentum (q_r) of the phonon is equal to the momentum transfer (q_m) measured by HREELS. However, if the sample surface is not flat enough, the roughness will produce small facets with different orientations. Correspondingly, the angle between the incident direction and the facet normal, i.e., the θ_i, varies with the facet orientations. Therefore, for a specific q_r, θ_s also varies with the facet orientations [Fig. S2c]. This means that the phononic scattering signal at a specific q_r contributed by many small facets will be replicated to various q_m. This produces the replica signals of phonons uniformly distributed in momentum space.

In general, the overall intensity of the replica signals contributed by the small facets is related to the roughness of the sample surface. The intensity distributions of the replica signals in the 2D HREELS spectra are affected by the following two factors:

1) Intrinsic phonon dispersion of materials. At the energy position where the slope of the phonon dispersion is very small, the phonon frequency is almost constant in a certain momentum range. This enables the replica signals in this momentum range to be superimposed, increasing the intensity of the replica signals.
2) Selection rules for HREELS. The selection rule for HREELS determines the scattering intensity of phonons. The phonons with greater scattering intensity will produce greater intensity of the replica signals.

In our measurements, the replica signals originating from small facets are all related to phonons with larger scattering intensity and smaller dispersion slope (Fig. S3), which is consistent with the above discussions. Furthermore, according to our calculations using Eq. (2), for a phonon mode at

$q_r = 1$ Å^{-1}, a facet with a roughness angle as small as 10° will make the replica signal cover the entire first Brillouin zone. Thus, the roughness of the Cu foil substrate can easily give rise to the replica signals throughout the 2D phonon spectra of h-BN measured by HREELS.

It should be emphasized that in our measurements of h-BN/Cu foil, there is a main scattering plane on the sample surface, which contributes most of the intensity of the scattered signal. Figure S4a shows the 2D mapping near the zero-loss energy along the Γ-K direction. There is a bright spot with the maximum scattering intensity around the position of zero energy loss and zero momentum in the 2D mapping, corresponding to the specular scattering of electrons from the main plane. To show the dependence of the scattering intensity on the momentum at zero loss energy, we extracted the momentum distribution curve (MDC) and displayed it in Fig. S4b. The extremely strong intensity of zero loss peak along the momentum direction indicates that most of the specular scattering signal comes from one main plane (specular scattering from other small facets contributes to a finite-momentum background in MDC). Correspondingly, most of the inelastic scattering signals in our measurements are also contributed by the main plane, which reflects the true phonon dispersions of h-BN. This is also demonstrated by the good agreement between our measured and calculated phonon dispersions. Therefore, the small scattering facets of the sample can only bring some replica signals and will not affect the real phonon dispersions from the scattering of the main plane.

Comparison of calculated optical phonon dispersions

Figure S5 compares the optical phonon dispersions calculated using 2D implementation and traditional 3D boundary periodic conditions. The calculated results using the 2D implementation effectively demonstrate the degeneracy of LO and TO phonons, as well as the “V-shaped” nonanalytic behavior of the LO phonon, which aligns with the experimental observations. Conversely, the 3D periodic boundary conditions consistently introduce spurious effects from the periodic images, resulting in a finite energy gap between LO and TO phonons, and “U-shaped” behavior of the LO phonons. This energy gap, while decreasing with increasing vacuum distance, never reaches zero, highlighting the long-range nature of the Coulomb interaction.

Supplemental 2D-HREELS data of h-BN grown on Cu single crystal

To clarify the special screening effect of Cu foil, we performed 2D-HREELS measurements of monolayer h-BN grown on Cu single crystal as a comparative experiment. Figure S6 shows the full phonon dispersion measurement of h-BN/Cu crystal. Due to the flat surface of Cu single crystal, the replica signals appearing in h-BN/Cu foil do not exist anymore (Fig. S6b), and the quality of phonon spectra is much better than the results of h-BN/Cu foil.

To make sure the reproducibility of the observed nonanalytic phonon behavior, we have performed the 2D-HREELS measurements on different h-BN/Cu foil and h-BN/Cu crystal samples. Fig. S7 demonstrates the results from other samples different from the ones used in the main

manuscript. All the measurements on different samples show reproducible and consistent results.

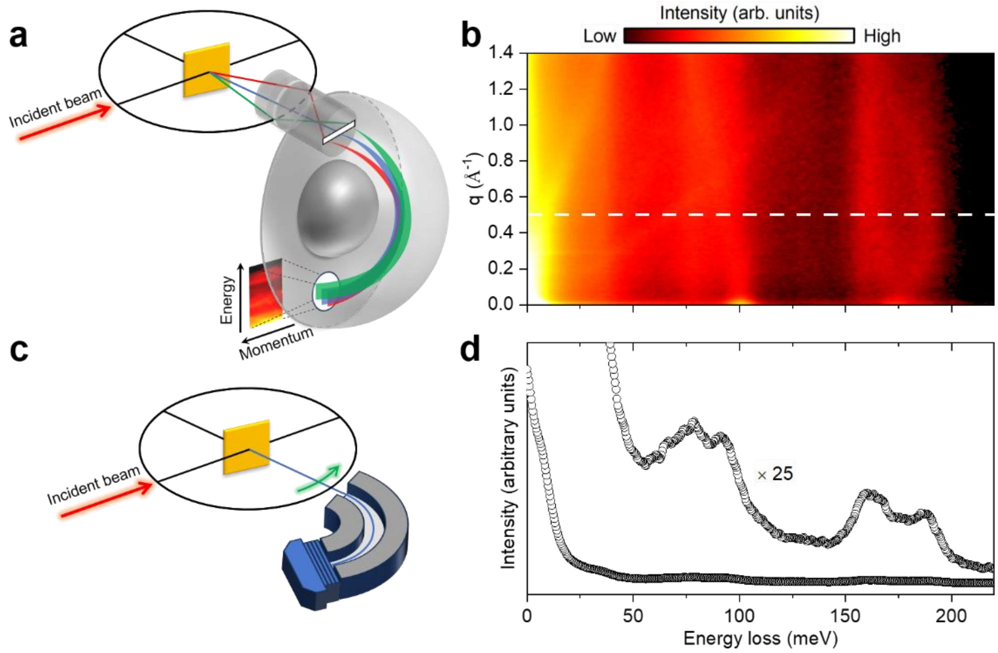

FIG. S1. Comparison of 2D-HREELS and conventional HREELS. **a** and **c**, Schematics of the 2D-HREELS and conventional HREELS setup, respectively. A schematic of the hemispherical electron energy analyzer is shown in **a**. **b** and **d**, the 2D energy-momentum spectrum and one-dimensional EDC obtained by single measurement of 2D-HREELS and conventional HREELS, respectively. The EDC in **d** corresponds to the white dashed line in **b**.

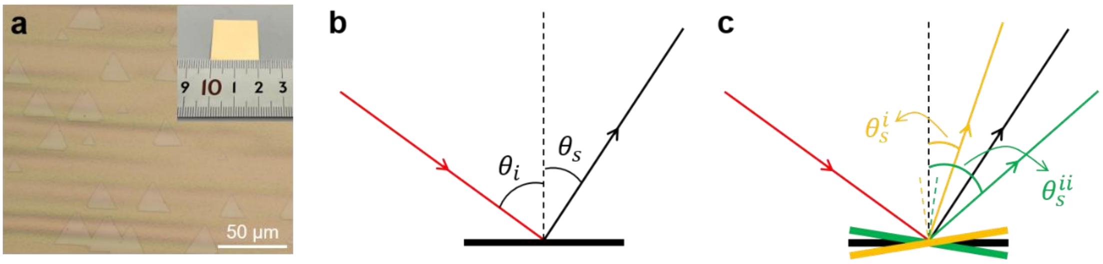

FIG. S2. Sample characteristic and HREELS scattering geometry. a, Optical image of sub-monolayer h-BN grown on Cu foil. The image shows apparent roughness caused by the cold rolling process. **b**, Schematic of the HREELS scattering geometry on a flat plane. **c**, Schematic of the HREELS scattering geometry for a specific q_r on different small facets.

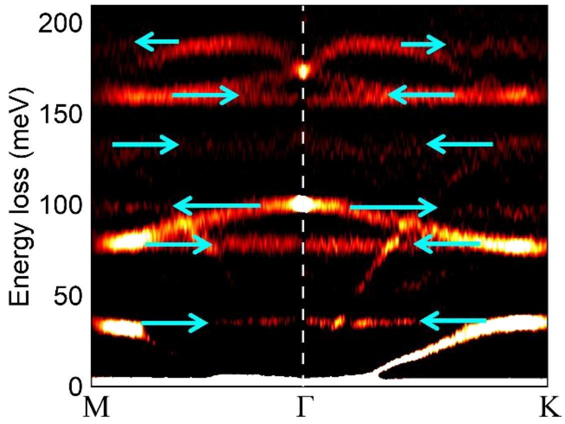

FIG. S3. Origin of replica signal in HREELS spectra. The bright blue arrows mark the phonon modes corresponding to the replica signals.

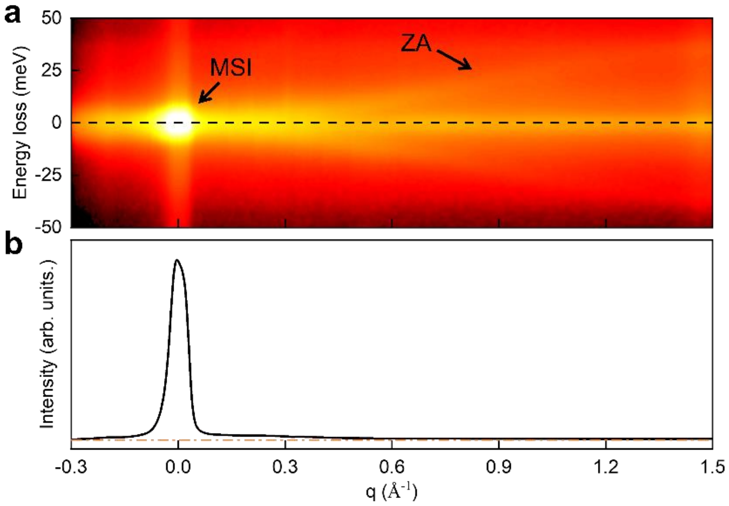

FIG. S4. Specular scattering at zero-loss energy of the monolayer h-BN/Cu foil. a, 2D mapping near the zero-loss energy along Γ-K direction. The position with the maximum scattering intensity is marked with MSI. **b**, MDC at the zero-loss energy [corresponding to the black dotted line in **a**].

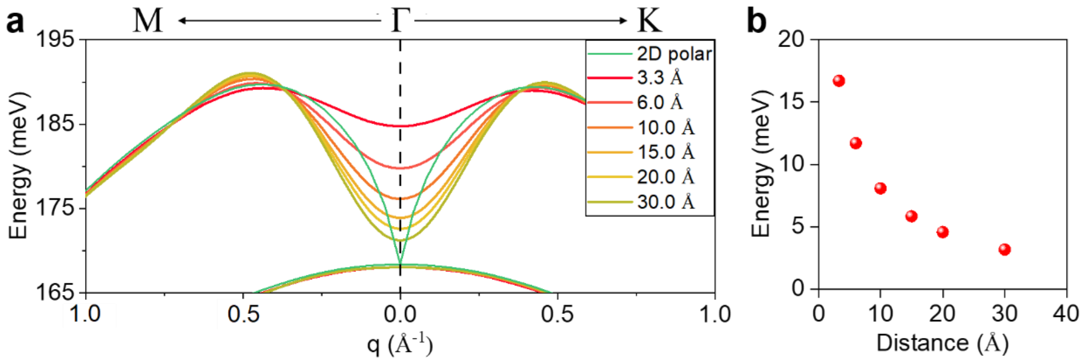

FIG. S5. Calculated LO and TO phonon dispersions of monolayer h-BN by DFPT. a, Results of 2D implementation (green curves) and traditional 3D boundary periodic conditions [with 3.3, 6.0, 10.0, 15.0, 20.0, and 30.0 Å of vacuum between periodic images (other curves)]. **b**, The energy difference between the LO and TO modes at the CBZ *vs* vacuum distance from traditional 3D treatment.

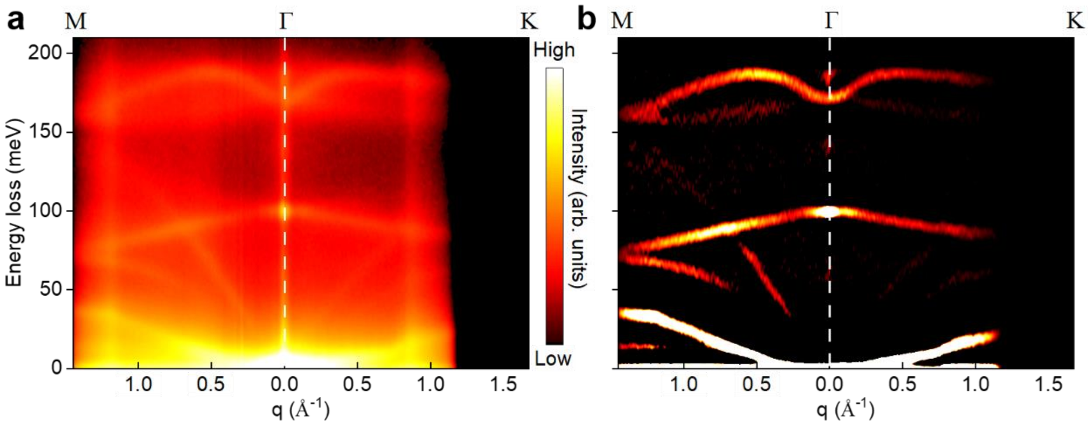

FIG. S6. Phonon spectra of monolayer h-BN/Cu crystal. a, 2D energy-momentum mappings of 2D-HREELS along the Γ–M and Γ–K directions. **b**, The second derivative results correspond to **a**. Due to the flat surface of Cu single crystal, the quality of phonon spectra is much better than the results of h-BN/Cu foil. In the second derivative spectra, a surface phonon of the Cu single crystal can be resolved near the M point (~ 14 meV), and all phonons have no obvious replica signals.

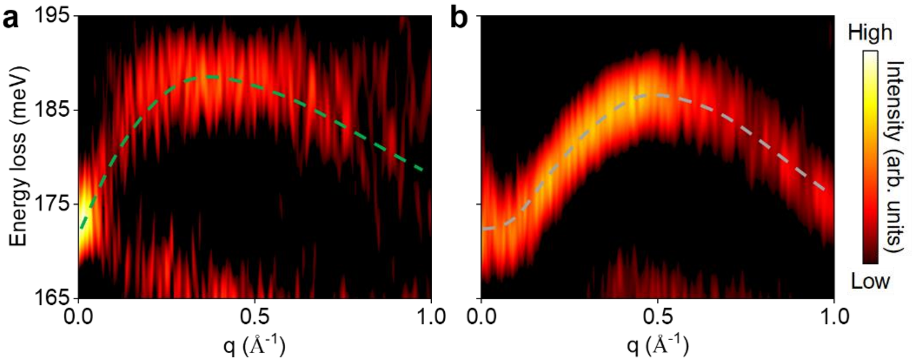

FIG. S7. Reproduction experiment of LO phonon behaviors on monolayer h-BN. a and **b** are the phonon spectra on another h-BN/Cu foil and h-BN/Cu crystal samples, respectively. The phonon spectrum shown in **b** demonstrates the absence of adsorption vibrations of carbon on this h-BN/Cu crystal sample, while the finite slope of LO phonon near the Γ point is still completely suppressed. This suggests that the adsorption of carbon does not have an impact on the experimental results.
